# Threat to the predator suppresses defence of its prey

**DOI:** 10.1098/rsos.241711

**Published:** 2025-01-22

**Authors:** Monika Sysiak, Piotr Maszczyk, Andrzej Mikulski

**Affiliations:** ^1^ Department of Hydrobiology, Institute of Ecology, Faculty of Biology, University of Warsaw, Żwirki i Wigury 101, Warsaw 02-089, Poland

**Keywords:** chemical cues, predation, odonata, *Daphnia*, fear cascades, cannibalism

## Abstract

Many studies have shown that prey can optimize their defence mechanisms based on cues indicating predator presence and pressure. However, little is known about whether prey can assess the actual threat by considering both predator density and the motivational state of cannibalistic predators, which can be influenced by threats from higher order predators. We conducted laboratory experiments to test the hypothesis that high predator density, combined with chemical cues indicating predator stress (e.g. alarm and disturbance cues), may inhibit prey defences. Using *Daphnia* and Zygoptera/Anisoptera larvae, we observed that *Daphnia’s* strong response to low-density predator kairomones was suppressed when exposed to high-density predator kairomones and disturbance cues. Surprisingly, we found no evidence of a suppressive response to alarm cues. Our study is to our knowledge, the first to show that prey uses predator stress cues to avoid unnecessary defences, suggesting a ‘cascade of fear’ in which fear at one trophic level reduces fear at a lower level. Furthermore, it is to our knowledge the first to demonstrate that prey can reduce their anti-predator response in the presence of high densities of cannibalistic predators.

## 1. Introduction

Death by predation is one of the strongest factors of natural selection. Not surprisingly, a complex system has evolved to protect organisms against this threat. The response to predation is often associated with fundamental changes in prey physiology, behaviour, life history and morphology [1]. As energetic costs associated with defence strategies are typically high, precise control over timing and strength of response is crucial for fitness [2,3]. Therefore, the evolution of anti-predator mechanisms has been accompanied by the evolution of threat assessment methods, enabling organisms to avoid the costs of unnecessary responses. The response must be precisely matched to the particular predator and the severity of its pressure. This adjustment requires a reliable and detailed signal on the nature of the threat and its precise interpretation by the prey [4].

In aquatic habitats, factors such as turbidity, habitat complexity, water flow and light conditions can impede visual perception, making chemical communication the most reliable means of obtaining information about predation risk [5,6]. Furthermore, its reliability is enhanced by the persistence of infochemical gradients in water with high viscosity compared to the turbulent atmosphere [7]. Among infochemicals, kairomones seem to be the most widespread carriers of information [2]. These semiochemicals—chemical cues that transmit information between individuals—mediate interspecific interactions that benefit the receiver rather than the emitter [8]. The ability of potential prey to detect such predator-related kairomones and phenotypically adjust their traits may enhance predator avoidance or resistance [9]. In addition to kairomones, there are other cues that inform prey about the threat of predation. One is alarm cues released by injured individuals, which alert other potential prey to the presence of a foraging predator [10]. Another is disturbance cues, which are substances released by an individual that has been approached by a predator but not injured during the encounter. In this way, disturbance cues alert other individuals to the presence of a hunting predator [11].

Cues associated with predators can convey substantial information about the level of risk, offering insights that extend far beyond merely indicating the presence or absence of a predator. One way to obtain quantitative information about the actual threat is through the prey’s ability to detect various types of predator cues [12], allowing them to integrate information from multiple sensory modalities to reduce uncertainty, and rapidly adjust their responses according to the perceived threat [13,14]. For example, light intensity influences the extent of life-history changes in cladocerans of the genus *Daphnia* caused by exposure to kairomones produced by visually oriented predators (e.g. fishes). This plasticity enables *Daphnia* to respond effectively to fluctuating environmental conditions while minimizing the associated costs of defence [15]. Another example involves predator-induced diapause in *Daphnia*, which requires the simultaneous presence of two chemical cues: kairomones and alarm cues [16].

**Figure 1.**
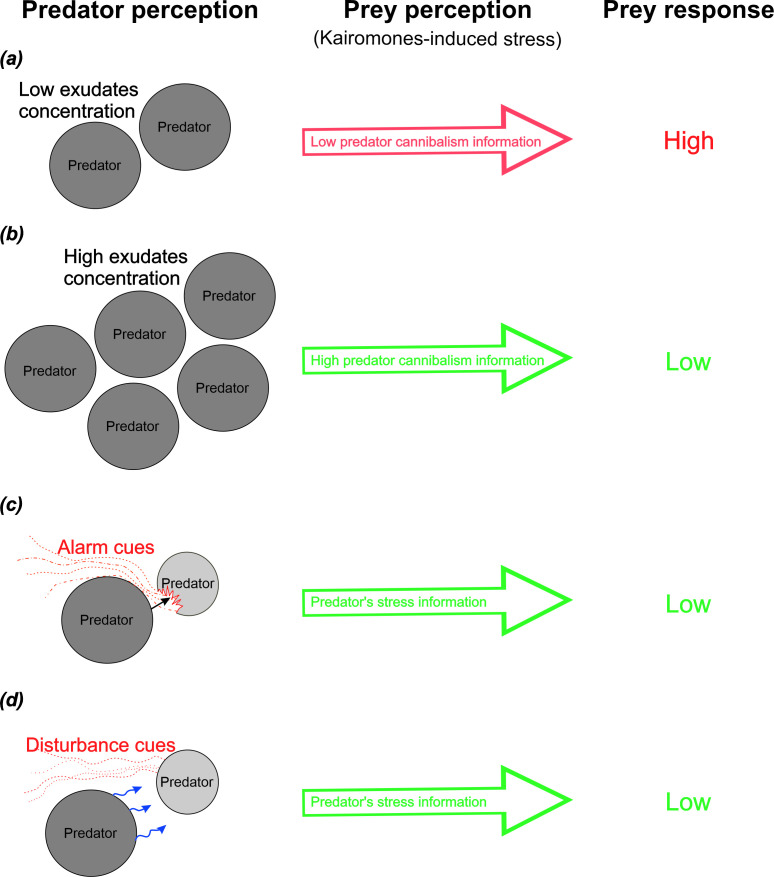
(*a*) Low risk of cannibalism for the predator—the predator perceives a low density of conspecifics and can actively hunt. The prey perceives a low concentration of predator kairomones, which informs it of increased predator pressure. The prey's defensive response is high. (*b*) High risk of cannibalism for the predator—the predator perceives a high density of conspecifics and cannot actively hunt. The prey perceives a high concentration of predator kairomones, which informs it of reduced predator pressure. The prey's defensive response is low. (*c*) Injured predator releases an alarm cue (AC)—other predators perceive information about danger and reduce activity. Prey perceives predator kairomones with AC, informing them of reduced predator pressure. The prey's defensive response is low. (*d*) Stressed predator releases a disturbance cue (DC)—other predators perceive danger information and reduce activity. The prey perceives predator kairomones with DC, informing it of reduced predator pressure. The prey's defensive response is low.

Another way to obtain quantitative information about the actual threat is through the prey’s ability to assess the intensity of a cue, which determines the magnitude of its response. In the case of chemical cues, prey can evaluate predator cues in a concentration-dependent manner, reflecting predator population density. This is clearly demonstrated in *Daphnia*, where the strength of their behavioural [17] and life history [18] of anti-predator defences is positively correlated with the concentration of fish kairomones [19]. However, a different scenario can be expected regarding prey responses to cues from cannibalistic predators. In populations of such predators, an individual may simultaneously become both predator and prey as a result of cannibalism. Thus, when individuals from cannibalistic populations receive information about the presence of conspecifics, it may be equivalent to receiving information about the presence of food or a threat [20,21]. Whether this positions them as perpetrators or prey of cannibalism, it may reduce their interest in foraging for other species. Typically, cannibalism increases as population densities rise [22]. This information could reach the prey through changes in kairomone concentrations. In this context, an increase in kairomone concentration would not lead to an increase in predator pressure, as is typically observed [17,18], but rather to a decrease (figure 1*a*,*b*). This is supported by a previous study, in which damselfly larvae (*Ischnura elegans*) exhibited reduced hunting activity when exposed to high concentrations of conspecific kairomones [23]. However, it remains unclear whether prey from other species can also estimate the population density of cannibalistic predators and respond accordingly to the density-dependent threat.

Furthermore, another way to obtain quantitative information about the actual threat is through the prey’s ability to assess cues related to the motivational state of predators, including their hunger level and the stress level from higher order predators. Numerous studies have shown that chemical cues are sufficient to provide reliable information to the prey about the predator’s diet [24–27]. The predator’s hunger state has been found to influence anti-predator responses, with chemical cues from food-deprived predatory fishes eliciting anti-predator behaviour in smaller prey fishes [28] and chemical cues from food-deprived predatory wolf spiders, such as *Hogna helluo*, triggering anti-predator behaviour in smaller wolf spiders like *Pardosa milvina* [29]. Much less is known about prey responses to chemical cues indicating that their predators perceive a threat from higher order predators. Potential prey that are able to identify chemical cues that alert their predator to danger and modify their phenotype accordingly should be favoured by natural selection. This allows them to avoid unnecessary defence costs that are inadequate to the situation. Thus, a chemical cue that informs predators of danger could be a signal of reduced danger for prey. This suggests the possibility of a kind of fear cascade, in which the fear sensed by individuals at one trophic level is translated into a reduction of fear at a lower level [30,31]. When the mesopredator (dotty back, *Pseudochromis fuscus*) was exposed to the acute risk from the top predator (visual cues of a coral trout, *Plectropomus leopardus*), its mobility was restricted, indirectly allowing a prey (juveniles of the damselfish *Pomacentrus amboinensis*) to reduce physiological stress [32]. This example has revealed the relationship between a predator’s perceived threat level and modifications in their prey’s response to the predator’s visual cues. However, there are no studies confirming that prey can adjust their behaviour by accurately interpreting the threat posed by predators based on the chemical cues they release. For instance, this mechanism could operate through the prey’s ability to receive and accurately interpret alarm cues released when a predator is injured, as well as predator disturbance cues that indicate predator stress ([10,11]; figure 1).

Odonate larvae serve as a convenient cannibalistic predator model for studying how their population density and stress induced by higher order predators affect prey responses. Previous studies have reported that odonate larvae are predators that implement defensive mechanisms reducing their hunting activity [33]. This suggests that they can assess predation risk based on chemical cues and adjust their foraging activity accordingly. The two odonate suborders (Anisoptera and Zygoptera) differ in the range of prey they can forage on and the predators that can hunt them. Comparing prey responses to signals from both groups of predators will therefore provide information on the prevalence of this phenomenon. Both suborders of odonate larvae typically feed on other insects or planktonic animals [34].

*Daphnia* appears to be an excellent model as prey for odonate larvae. The relatively large size range during ontogeny makes *Daphnia* attractive prey for many predators [35]. *Daphnia* are filter feeders that consume small particles suspended in the water by using a filtering apparatus—a sieve created by filter combs on their filtering trunk limbs. The limbs of the filtering trunk move rhythmically, causing water to flow between them and the carapace. Thus, the faster *Daphnia* moves, the faster its filtering trunk limbs work, resulting in more water being filtered and an increased amount of food particles ingested [36,37]. Because of the direct link between food acquisition and locomotion in *Daphnia*, the filtration rate (the removal of food particles from a given volume of water per unit of time [38]) is also a good parameter for studying their defence mechanisms against predators. These mechanisms may include behavioural changes, such as escape [39], or a decrease in locomotor activity caused by the presence of predator chemical cues. For example, *Daphnia pulex* reduces locomotor activity in the presence of *Chaoborus* larvae, resulting in a decrease in filtration rate [40]. This reduction in the frequency of filtering trunk limb movements decreases the chances of detection by predators [41]. Changes in *Daphnia* filtration rates are therefore an appropriate trait to observe how chemical cues about threats from predators alter the adaptive response of prey.

We conducted an experiment to measure the filtration rate as a proxy of the defensive response of *Daphnia magna* during exposure to chemical cues from cannibalistic predators, either Zygoptera larvae (*Ischnura elegans*) or Anisoptera larvae (*Brachytron pratense*). *Daphnia* were exposed to information on low and high odonate population density, as well as information indicating their stress (alarm cues and disturbance cues) from higher order predators (larger bodied odonate individuals). We hypothesized that *Daphnia* would exhibit a strong response to the presence of kairomones from odonate larvae at low population density (figure 1). However, this response would be suppressed when *Daphnia* were exposed to kairomones from predators at high density (figure 1), as well as when they were subjected to predator alarm and disturbance cues (figure 1).

## 2. Material and methods

### 2.1. Experimental organisms

#### 2.1.1. Origin of *Daphnia*, damselflies and dragonflies

Three clones of *D. magna* (N, B and W) were established from hatched ephippial eggs originating from different water bodies where *Daphnia* coexist with fishes and odonate predators. Clone N was sourced from Novy Vrbenský Rybník pond, which is part of a complex of eutrophic ponds within the Vrbenský Rybník reserve, located near České Budějovice in the Czech Republic (49°00′33.4″ N, 14°26′40.9″ E) [42]. Clone B was sourced from Großer Binnensee, situated in northern Germany (54°19'37.8" N, 10°37′35.1″ E), a hypertrophic lake that periodically receives saltwater from the Baltic Sea [43]. Clone W was sourced from the Żabieniec Ponds, a system of fish ponds that are drained in winter, located near Warsaw, Poland (52°03′01.9″ N, 21°01′48.4″ E).

Damselfly larvae, *I. elegans* were collected from Lake Czerniakowskie in Warsaw, Poland (52°11′16.4″ N, 21°04′28.3″ E)—a natural lake in the city inhabited by fishes and invertebrate predators.

Dragonfly larvae *B. pratense* were collected from Czarna Struga—river floodplain in northeastern Poland (53°41′58.5″ N, 21°55′38.8″ E), densely overgrown with macrophytes and inhabited by various invertebrates and fish predators.

#### 2.1.2. Culture of *Daphnia* and maintenance of damselflies and dragonflies

All *Daphnia* clones, as well as damselfly and dragonfly larvae, were kept in the laboratory of the Department of Hydrobiology, Faculty of Biology, University of Warsaw. The laboratory was a temperature-controlled room maintained at 20.0 ± 0.5°C, with a photoperiod of 16 L : 8 D.

Each *Daphnia* clone was cultured in eight, 1 l glass vessels (10 individuals in each), containing conditioned and filtered (1 µm filter) lake water from the city pond Glinianki Szczęśliwickie in Warsaw Poland (52°12′25.1″ N, 20°57′36.1″ E). The water was aerated for at least two weeks prior to use. Individuals were fed daily with 1 mg carbon (C_org_) per litre of the algae *Chlamydomonas* sp. The water was changed every other day. To maintain a stable temperature, the vessels were placed in a water bath with a controlled temperature of 20.0 ± 0.3°C. To ensure that non-genetic intergenerational transmission of information did not differentiate individuals [44], each clone was initially maintained in the laboratory for three months under identical conditions. Subsequently, prior to the experiments, the clones were kept for three generations under the same conditions as those in the experiment. Each generation was established from the second clutch of the previous generation.

Before the preparation of experimental media, damselfly and dragonfly larvae were photographed and measured using NIS-Elements BR 3.2 software. Individuals of *I. elegans* were assigned to instars based on measurements of body length and head width [45]. Additionally, instar assignment was confirmed by visual inspection of the stage of wing bud development. In the experiments, two groups of individuals were used: (i) cannibals—12th instar, and (ii) prey of cannibals—7th instar. *Brachytron pratense* larvae were classified into specific size groups based on the assessment of developmental stages and measurements. The ranges for each parameter were determined as follows: (i) cannibals: body length 11–12.42 mm, head width 4.54–4.79 mm, wing bud development 6.75–7.75 mm; and (ii) prey of cannibals: body length 3.08–4.42 mm, head width 1.7–1.9 mm, wing bud development 1.75–2.75 mm. Additionally, since injuries may affect larval behaviour, all damselflies and dragonflies were checked for signs of mechanical damage, such as the loss of lamellae or legs. Before the preparation of experimental media, all larvae were cultivated separately (to prevent cannibalism) in containers filled with 200 ml of conditioned, constantly aerated lake water and fed daily with 20 individuals of *Daphnia*.

### 2.2. Experimental system

The experiment was conducted in the same room as the culture. The experimental set-up included a plankton wheel to prevent sedimentation of algal cells [46]. The wheel was placed in a well-isolated box (120 × 106 × 56 cm) with an internal temperature of 20°C. The set-up was equipped with two LED lamps (LED SYSTEMS, C70-LT504-30, 4W) covered with a dark diffuser to ensure uniform and low light intensity (11.1 ± 0.3 μmol m⁻² s⁻¹, measured with a Li-COR 250A photometer) and to limit algal growth during the exposure period. Lighting was necessary because *Daphnia* may not respond to the kairomones of a visual predator in the dark [39].

### 2.3. Experimental design

#### 2.3.1. General information

In total, six experiments were conducted, each using a different clone of *Daphnia* exposed to either damselfly or dragonfly larvae cues. In each experiment, 10 *Daphnia* individuals in the pre-adult instar were used for each of the five different experimental media, placed individually in separate 15 ml Falcon tubes. An additional 15 tubes served as controls, with three tubes assigned to each of the five media without animals.

#### 2.3.2. Media preparation

In accordance with previous study [23], which showed that a high concentration of conspecific kairomones from *I. elegans* was more stressful for larvae than low concentrations (5 and 2 individuals l^−1^, respectively), we hypothesized that the stress experienced by *Daphnia* (as indicated by changes in filtration rate) would be greater when exposed to cues indicating low densities of *I. elegans* or *B. pratense* compared to high densities (2 and 5 individuals l^−1^ respectively).

The media used in the experiments were as follows: (i) control medium (C) (without any predator signal); (ii) medium (L) with information on the low density of a predator (*I. elegans* or *B. pratense*) (2 individuals l^−1^); (iii) medium (H) with information on high density of a predator (5 individuals l^−1^); (iv) medium (L + AC) with information on low density of a predator and predator alarm cues (chemicals released by injured conspecifics); and (v) medium (L + DC) with information on low density of a predator and predator disturbance cues (chemicals released by stressed predators). Different individuals of damselfly or dragonfly larvae designated as ‘cannibals’ were used to prepare each of the experimental media in each experiment.

The control medium (C) contained only conditioned and filtered lake water (0.2 µm filter) placed in three 1 l glass vessels. The same water was used in the remaining media. Media L and H were prepared by placing three starved predators (either *I. elegans* or *B. pratense*) separately in vessels filled with 500 and 200 ml of water, respectively, 24 h before the experiments. To create media with different odonate densities, we used different volumes of water with single individuals rather than varying the number of individuals in the same volume. This approach was taken to eliminate any competitive or cannibalistic interactions that might otherwise arise between individuals. Odonates were not fed for two days prior to media preparation to avoid contaminating their chemical cue with food items that could potentially serve as a source of prey alarm cues. The medium L + AC was prepared similarly to medium L, with one difference: the small larva (prey of cannibals) was placed in a vessel with an individual producing information about low densities of odonates. An additional larva was only placed in each vessel 2 h prior to the experiment. This resulted in a one-to-one interaction in which the smaller larva was cannibalized, leading to the emission of fresh alarm cues by the cannibalized individual. It was assumed that the presence of the small larva for 2 h did not significantly increase the information about the density of the odonates in the medium. The medium L + DC was prepared similarly to medium L, with the exception that the disturbance cues were generated by stressing odonate larvae in each vessel for 90 min before the experiment. The stress was induced by automatically lowering a frame with rods ending in streamlined rubber caps, causing the caps to plunge violently into the water every 10 min, simulating odonate attacks. Media from all vessels used for a particular treatment were filtered through a 0.2 μm antibacterial filter just before the experiment, and 1.0 mg of organic C_org_ from *Chlamydomonas* sp. was added.

#### 2.3.3. *Daphnia* stress measurements—filtration rate

In each experiment, media from each treatment were first added randomly to each of the 65 Falcon tubes. To prevent uneven food distribution, the continuously stirred medium was pipetted into the tubes in three series of 5 ml. Subsequently, 50 Falcons were individually inoculated with *Daphnia* (10 *Daphnia* for each treatment). All 65 Falcons were randomly placed in 1 l glass vessels filled with 20°C distilled water. Finally, the vessels were closed and placed on a rotating plankton wheel.

As the kairomones degrade over time [47], which may alter their effects on the animals and confound differences between treatments, and because the response of *Daphnia* to chemical cues is typically most pronounced early in the exposure phase and declines over the first few hours [48], we limited the time period for examining filtration rates to 3 h. We measured algal cell loss using a Turner Aquafluor Handheld Fluorometer. *Daphnia* lengths were measured using NIS-Elements BR 3.2 software. The final result was obtained by subtracting the algal concentration in the tubes with animals from that in the corresponding tubes without animals (using the average of three blank tubes for each medium). Consumption was then converted to milligrams of C_org_ per hour according to formula:


$$\text{FR}=\;\frac{V\left(C_{0\;}-\;C_{1}\right)}{t},$$

where FR is the filtration rate; *V* is the volume of the chamber; *C*_0_ is the organic carbon concentration at the beginning of the experiment; *C*_1_ is the organic carbon concentration at the end of the experiment and *t* is the time.

### 2.4. Data analyses

Statistical analysis was performed using Statistix 9.0. The Shapiro–Wilk test was employed to assess the normality of the data, while homogeneity of variance was evaluated using Levene’s test [49]. The significance of the effect of the presence of chemical information on threat (C—control, L—information on low density of odonates, H—information on high density of odonates, L + AC—information on low density of odonates with alarm cues, L + DC—information on low density of odonates with disturbance cues), as well as *Daphnia* clonal affiliation (clones B, N and W), and the taxonomic position of odonates larvae (Anisoptera and Zygoptera) on *Daphnia* filtration rate was analysed by three-way ANCOVA with *Daphnia* size as a covariate. *Post-hoc* comparisons of filtration rates between individuals exposed to different chemical cues (both overall and for individual clones) were performed using Tukey’s HSD test at a significance level of *p* < 0.05. Four observations were automatically excluded from the analysis owing to incomplete data.

## 3. Results

### 3.1. General ANCOVA

The general ANCOVA showed a significant effect of chemical cues and the effect of clones on the filtration rate of *Daphnia*. The effect of the dragonfly suborder was not significant (table 1). However, all interactions between the effects of all the factors studied were significant.

**Table 1.** Results of the three-way ANCOVA examining the effects of chemical cues (C, H, L, L + AC and L +DC), clone (N, B and W) and the predator's taxonomic position (damselfly and dragonfly), and their interactions on the filtration rate of *Daphnia*, with *Daphnia* size as a covariate.

effect	d.f.	*F*	*p*
treatment	4	16.34	<0.00001
clone	2	77.69	<0.00001
suborder	1	0.00	0.98760
treatment × clone	8	2.06	0.03990
treatment × suborder	4	5.41	0.00030
clone × suborder	2	4.17	0.01650
treatment × clone × suborder	8	9.55	<0.00001
size (covariate)	1	54.90	<0.00001
error	265		
total	295		

### 3.2. Anisoptera as predators

**Figure 2.**
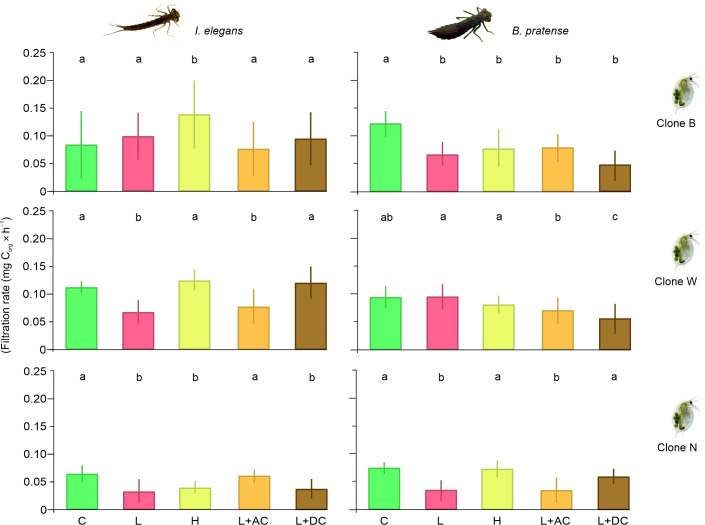
Filtration rate (mean ± 1 s.d.) of each of the *D. magna* clones (N, B and W) exposed to chemical cues from Zygoptera and Anisoptera larvae: control medium (C); information on low density of a predator (2 odonates l^−1^) (L); information on high density of a predator (5 odonates l^−1^) (H); information on low density of a predator and predator alarm cues (L + AC); and information on low density of a predator and predator disturbance cues (L + DC). Treatments that were significantly different from the others are denoted with different letters.

The filtration rate for individuals from clone B was significantly lower across all cue combinations (H, L, L + AC and L + AD) compared to the control group (figure 2). For clone N, the filtration rate significantly decreased in response to cues for low odonate densities (L) and low odonate densities with alarm cues (L + AC) compared to the control (figure 2). Additionally, clone N’s filtration rate was significantly lower in the L treatment compared to the H and L + DC treatments, and in the L + AC treatment compared to the H and L + DC treatments (figure 2). For clone W, the filtration rate was significantly reduced in the following cases: (i) in the L + AC and L + DC treatments compared to the control; (ii) in the L + DC treatment compared to the L + AC treatment; and (iii) in the L + AC and L + DC treatments compared to the L treatment (figure 2).

### 3.3. Zygoptera as predators

The filtration rate for individuals from clone B was significantly higher in the H treatment compared to the remaining treatments (control group, L, L + AC and L + AD; figure 2). For clone N, the filtration rate was significantly lower in the L, H and L + DC treatments compared to the control group and the L + AC treatment (figure 2). For clone W, the filtration rate was lower in the L and L +AC treatments compared to the remaining three treatments (control group, H and L + DC; figure 2).

## 4. Discussion

The general model encompassing all *Daphnia* clones and both dragonfly and damselfly larvae revealed a strong interaction between the factors. Responses to different types of threat information were interpreted differently by individual clones. Owing to the varied directions of these responses among clones, there is potential for significant error in drawing conclusions about general trends. Consequently, we focussed on analysing the data for each *Daphnia* clone and type of odonate larvae individually. These analyses demonstrated that information about predator density served as a more reliable signal for *Daphnia* than other types of cues. Density-related responses were more frequent and generally stronger compared to those triggered by other cues, as observed in the cases of clone W in response to *I. elegans* and clone N in response to *B. pratense*. While these responses exhibit variability, this method reveals fairly consistent patterns within the data. Specifically, information about low densities of odonate larvae increased *Daphnia* alertness in four out of six cases, while high densities decreased alertness in three cases. These findings indicate that *Daphnia* adjust their responses based on predator density, which appears to be an adaptive strategy. The contrasting response to cannibalistic predators compared to non-cannibalistic ones, such as fishes, suggests that *Daphnia* face a trade-off in balancing mortality risk between these two predator types. Additionally, the results imply that disturbance cues from predators may signal a reduced threat for *Daphnia*, although this effect was evident in only two out of six cases (clone W in response to *I. elegans* and clone B in response to *B. pratense*). A similar mechanism enabling prey to recognize a predator’s fear could be common, as conspecific interactions are critical for many predatory invertebrates [50–52], and their likelihood can significantly limit foraging activity [20,23]. However, our findings did not conclusively show that *Daphnia* suppress their response to predator alarm cues, as this was only observed in one case, with another showing an inverted response pattern. A potential explanation for the negligible effect of alarm cues is that it may have been obscured by the influence of information contained in the exudates produced by the cannibalistic predator during prey consumption. However, this explanation seems unlikely, as exudates from cannibalistic predators typically convey information about increasing predator density, which, according to our results, should amplify rather than diminish the effects induced by alarm cues. To address uncertainties regarding the negligible effect of predator alarm cues on *Daphnia*, future studies could investigate this phenomenon using alarm cues derived from crushed odonate larvae instead of those generated during direct interactions between cannibalistic odonate larvae and their prey. Another concern with our method of obtaining alarm cues is that it may have introduced variability in cue reliability, potentially leading to differences in *Daphnia* responses across experiments. This variability might arise because the odonate prey could have been injured differently or at different times during their exposure to the predator in each experiment. However, we believe that the source of this variation was probably minimal, as *Daphnia* were exposed to a mixture of alarm cues derived from separate media in which individual cannibalistic odonates attacked their prey.

Some of the results in our study are ambiguous. For example, *Daphnia* clone B showed higher filtration rates in response to cues from high Zygoptera odonate densities compared to the control group, which lacked predator cues. One possible explanation is that high odonate densities might release not only kairomones but also nutrients, promoting bacterial growth in the medium and providing *Daphnia* with additional food, thus increasing filtration rates [53]. However, this is unlikely since the medium was filtered through 0.2 µm filters to minimize bacterial presence, and the experiment lasted only 3 h—too short for significant bacterial growth to affect filtration. The great inter-clonal variability in response is consistent with earlier observations that clones of the same *Daphnia* species may significantly differ in behaviour [54,55], life-history traits [56,57] and physiological traits [58,59] in response to single cues. Furthermore, this variability can be even more pronounced in the presence of multiple cues, where additional trade-offs influence the final outcome of the response [60].

The clonal variability observed in our study reveals additional hypotheses worth testing in the future. It may reflect adaptations to the distinct environmental conditions from which the clones originated, particularly variations in predation pressure from odonate larvae and differences in the relative activity of Anisoptera and Zygoptera. The significance of the interaction between clone and predator suborder in our results further underlines this relationship (table 1). Zygoptera and Anisoptera larvae differ in their environmental requirements, with Zygoptera appearing to be more niche dependent and Anisoptera showing greater ecological flexibility, which may also lead to differences in their abundance in a given pond [61,62]. Furthermore, Anisoptera can inhibit the colonization of Zygoptera in a shared pond [63]. This suggests that the predation pressure exerted by these predators on prey in the same pond may differ and could hypothetically result in different prey responses to their chemical cues. For example, the marked reduction in *Daphnia* foraging in response to both low and high densities of Anisoptera cues seen in *Daphnia* clone B, compared to other clones, could result from high predation pressure by Anisoptera in their native environment. This aligns with previous findings that a stronger antipredation response correlates with higher predation pressure [64,65]. Furthermore, the negligible effect of Zygoptera cues, paired with the strong response to Anisoptera cues in clone B suggests that Anisoptera are more prevalent than Zygoptera in their habitat. The additional source of variability in the obtained results is owing to the distinctly lower filtration rate of *Daphnia* clone N across all treatments compared to the other clones. This difference is most likely attributed to the slightly smaller body size of clone N individuals, despite the fact that *Daphnia* from all clones used in the experiments were of the same age. However, these are merely conjectures, as our experiment was not primarily designed to test this hypothesis. To better understand the clonal variation in response to the cues tested, it is necessary to study more clones, explore a wider range of chemical cue concentrations, and conduct a deeper investigation of trophic relationships within their native environments.

In conclusion, our research demonstrates for the first time to our knowledge, that prey can identify chemical cues indicating predator stress, which in turn signals a reduction in predator feeding efficiency, allowing them to enhance their fitness. This finding suggests the existence of a ‘cascade of fear’, where fear at one trophic level leads to reduced fear at a lower level. Furthermore, the study is, to our knowledge, the first to show that prey increase their anti-predator responses when exposed to low densities of cannibalistic predators, while reducing their responses at high predator densities. Thus, the study reveals that the density of cannibalistic predators can have broader implications, affecting a wider range of receivers beyond just conspecifics. Overall, these results provide valuable insights into our understanding of chemical communication at the predator−prey interface and lay the groundwork for future investigations in this field.

## Data Availability

Data are available as supporting Information: https://figshare.com/s/aea6ec05349bf24b985f.
